# *Clostridium difficile* in Ready-to-Eat Salads, Scotland

**DOI:** 10.3201/eid1505.081186

**Published:** 2009-05

**Authors:** Marwah M. Bakri, Derek J. Brown, John P. Butcher, Alistair D. Sutherland

**Affiliations:** Glasgow Caledonian University, Glasgow, Scotland, UK (M.M. Bakri, J.P. Butcher, A.D. Sutherland); Scottish *Salmonella*, *Shigella* and *Clostridium difficile* Reference Laboratory, Glasgow (D.J. Brown)

**Keywords:** Enteric infections, bacteria, antimicrobial resistance, Clostridium difficile, epidemiology, salad, environmental sources, foodborne, Scotland, dispatch

## Abstract

Of 40 ready-to-eat salads, 3 (7.5%) were positive for *Clostridium difficile* by PCR. Two isolates were PCR ribotype 017 (toxin A–, B+), and 1 was PCR ribotype 001. Isolates were susceptible to vancomycin and metronidazole but variably resistant to other antimicrobial drugs. Ready-to-eat salads may be potential sources for virulent *C. difficile*.

Over the past decade, *Clostridium difficile* infection has become a prominent cause of healthcare-associated infection. Although *C. difficile* has been thought of traditionally as a predominantly nosocomial infection, the incidence of community-acquired cases has increased recently, as has the incidence of cases from other healthcare settings such as nursing homes (*1*). Notably, some evidence has shown that *C. difficile* may be brought into the healthcare environment by asymptomatic carriers (*2*). The reported carriage rates of *C. difficile* in healthy adults have varied from 0% to 3% in Europe to up to 15% in Japan (*3*). Little is known, however, about the prevalence of *C. difficile* in the environment and how it may be transmitted to humans.

*C. difficile* has been found in a variety of environments, including water, soil, animal feces, and foods (*4*,*5*); these findings suggest that *C. difficile* may be transmitted to humans through food, although no foodborne cases have been reported. Because ready-to-eat foods have been implicated in foodborne disease outbreaks associated with *Salmonella* species (*6*) and *Escherichia coli* O157 (*7*), we examined ready-to-eat salads for the presence of *C. difficile*.

## The Study

We tested 50-g samples from each of 40 packaged ready-to-eat salads purchased from 7 Glasgow supermarkets from May 1 through June 30, 2008, for the presence of *C. difficile* spores. We essentially used the CDMN (*C. difficile*, moxalactam, norfloxacin) agar method of Rodriguez-Palacios et al. (*4*) but also used direct plating and enrichment broth culture. The contents of the 40 salads generally differed, and any salads with the same contents carried different supermarket brands, which eliminated replicate sampling.

Isolates were identified as toxigenic (having genes for toxins A and B) by PCR as previously described (*8*,*9*), and ribotypes were identified by PCR (*10*). The MICs of 6 antimicrobial drugs for these isolates were determined by using E-test strips (AB Biodisk, Solna, Sweden). The following MIC breakpoints were used to define resistance to these drugs: metronidazole, >32 µg/mL; vancomycin, >16 µg/mL; cefotaxime, 64 µg/mL; erythromycin, >8 µg/mL; moxifloxacin, >8 µg/mL; and clindamycin, >8 µg/mL (Clinical and Laboratory Standards Institute, Wayne, PA, USA).

*C. difficile* spores were detected in 3 (7.5%) of the 40 salad samples after culturing in enrichment broth (Table 1). Thirty-five (87.5%) of the salads were marked as imported from European Union countries; the remaining 5 were from UK suppliers. The 3 contaminated salads were not of UK origin. The 3 isolates were found to be toxinogenic by PCR; 2 were PCR ribotype 017, and 1 was PCR ribotype 001 (Table 1).

**Table 1:** PCR profile for toxins A and B and PCR ribotype of *Clostridium difficile* isolates, Scotland, 2008

Sample no./description	Toxin A	Toxin B	PCR ribotype
13/baby leaf spinach	–	+	017
24/organic mixed leaf salad	–	+	017
35/organic lettuce	+	+	001

The MICs of 6 antimicrobial drugs for each isolate and the resistance profile of each isolate are shown in Table 2. None of the isolates was resistant to vancomycin or metronidazole, and only the 001 isolate was resistant to moxifloxacin and erythromycin. All 3 isolates were resistant or intermediately resistant to clindamycin and cefotaxime; breakpoints for these drugs were highest for the 001 isolate.

**Table 2:** Susceptibility of 3 *Clostridium difficile* isolates to 6 antimicrobial drugs, by source of isolate, Scotland, UK, 2008*

Antimicrobial drug	Source of isolate		
	Baby leaf spinach (MIC, µg/mL)	Organic mixed leaf salad (MIC, µg/mL)	Organic lettuce (MIC, µg/mL)
Metronidazole	S (0.125)	S (0.094)	S (0.75)
Vancomycin	S (0.50)	S (0.38)	S (1.0)
Moxifloxacin	S (0.50)	S (0.75)	R (256)
Clindamycin	I (4.0)	I (6.0)	R (8.0)
Erythromycin	S (1.5)	S (0.75)	R (192)
Cefotaxime	I (48)	R (64)	R (256)

*S, sensitive; R, resistant; I, intermediate.

Isolates were obtained after being cultured in enrichment broth and not by direct plating, which suggests that spore counts were low (<3.0 CFU/g). The infectious dose required to colonize the healthy human gut is, however, unknown. Isolates were of PCR ribotypes 001 (a common clinical isolate in Scotland [*11*]) and 017 (a common European PCR ribotype containing isolates that are negative for toxin A and positive for toxin B [*12*]). No isolate was resistant to vancomycin or metronidazole, which is in accord with findings for other *C. difficile* isolates found in Scotland (*11*), but recent studies have highlighted the emergence of increased resistance to metronidazole among *C. difficile* isolates in England (*13*).

In general, the PCR ribotype 001 isolate was more drug resistant than the 017 isolates; it was the only isolate resistant to moxifloxacin and erythromycin and had the highest breakpoints to clindamycin and cefotaxime. In a 2005 study in which 271 *C. difficile* isolates from the UK were examined, all were found to be resistant to cefotaxime (*14*).

## Conclusions

The isolation of these PCR ribotypes from ready-to-eat salads is of concern and highlights the potential risk associated with consuming these salads, particularly since they are not cooked before being consumed. The consumption of these foods by vulnerable groups could possibly lead to *C. difficile* colonization and an increase in the asymptomatic *C. difficile* carriage rate among humans, thus increasing the risk for *C. difficile* transference within the healthcare environment (*2*). The presence of *C. difficile* in ready-to-eat salads could result from environmental contamination or transmission by food handlers. Further work is needed to investigate foods as a source of this pathogen and also to assess the role of soil and animals as its reservoirs.
